# Impact of cell geometry, cellular uptake region, and tumour morphology on ^225^Ac and ^177^Lu dose distributions in prostate cancer

**DOI:** 10.1186/s40658-024-00700-9

**Published:** 2024-11-21

**Authors:** Cassandra Miller, Ivan Klyuzhin, Guillaume Chaussé, Julia Brosch-Lenz, Helena Koniar, Kuangyu Shi, Arman Rahmim, Carlos Uribe

**Affiliations:** 1 Department of Integrative Oncology, BC Cancer Research Institute, Vancouver, BC Canada; 2 Department of Physics, University of British Columbia, Vancouver, BC Canada; 3 Department of Radiology, McGill University, Montreal, QC Canada; 4 Department of Nuclear Medicine, Bern University Hospital, The University of Bern, Bern, Switzerland; 5 Department of Radiology, University of British Columbia, Vancouver, BC Canada; 6 Molecular Imaging and Therapy, BC Cancer Research Institute, Vancouver, BC Canada

**Keywords:** Dosimetry, Monte Carlo simulations, Radiopharmaceutical therapy, PSMA

## Abstract

**Background:**

Radiopharmaceutical therapy with ^225^Ac- and ^177^Lu-PSMA has shown promising results for the treatment of prostate cancer. However, the distinct physical properties of alpha and beta radiation elicit varying cellular responses, which could be influenced by factors such as tumour morphology. In this study, we use simulations to examine how cell geometry, region of pharmaceutical uptake within the cell to model different internalization fractions, and the presence of tumour hypoxia and necrosis impact nucleus absorbed doses and dose heterogeneity with ^225^Ac and ^177^Lu. We also develop nucleus absorbed dose kernels for application to autoradiography images.

**Methods:**

We used the GATE Monte Carlo software to simulate three geometries of LNCaP prostate cancer cells (spherical, cubic, and ovoid) with activity of ^225^Ac or ^177^Lu internalized in the cytoplasm or bound to the extracellular membrane. Nucleus S-values were calculated for each geometry, source region, and isotope. The cell models were used to create nucleus absorbed dose kernels for each source region describing the dose to each nucleus in a cell layer, which were applied to simulated tumours composed of normoxic, hypoxic, or necrotic cancer cells to obtain dose rate maps. Absorbed doses within the tumours and dose heterogeneity were analyzed for each tumour morphology and isotope. Cell geometry made a minimal impact on S-values to the nucleus, however internalization resulted in higher nucleus doses. Applying the kernels to the simulated tumour maps showed that doses to each cell type varied between ^225^Ac and ^177^Lu depending on tumour morphology. Dose heterogeneity within tumours was slightly higher with ^225^Ac, however the tumour morphology made a larger impact on dose heterogeneity compared to the choice of isotope, with hypoxic and necrotic tumours having very heterogeneous dose distributions.

**Conclusions:**

Cell geometry simplifications may still allow robust results in simulation studies. Furthermore, the morphology of the tumour itself may make a larger impact on treatment response compared to other variables such as ratio of internalization. Finally, nucleus absorbed dose kernels were created that could enable microdosimetric studies with autoradiography.

**Supplementary Information:**

The online version contains supplementary material available at 10.1186/s40658-024-00700-9.

## Introduction

Radiopharmaceutical therapy (RPT), which involves the administration of cancer cell binding pharmaceuticals labelled with alpha, beta minus, or Auger-Meitner electron emitting radionuclides, has shown promising results in treating prostate cancer due to its ability to target cancer cells while minimizing toxicity to healthy tissues [1]. Lutetium-177 (^177^Lu) labelled prostate-specific membrane antigen (PSMA-617) ([^177^Lu]Lu-PSMA-617), was approved by the U.S. Food and Drug Administration (FDA) as Pluvicto™ in March 2022 [2] due to the therapeutic efficacy of ^177^Lu’s beta emissions and the high PSMA expression in metastatic castration resistant prostate cancer (mCRPC) [1]. PSMA binding radiopharmaceuticals labelled with alpha emitting radionuclides such as actinium-225 (^225^Ac) have also been shown to be effective with minimal toxicity and may result in superior treatment of mCRPC compared to beta emitters due to the high linear energy transfer (LET) of alpha particles [3].

^177^Lu emits beta minus particles and internal conversion electrons with mean and maximum ranges of 0.28 mm and 2.0 mm in tissue respectively [4, 5]. Conversely, the ^225^Ac decay chain emits four alpha particles with tissue ranges of 47–85 µm [5]. After binding to the prostate cancer cell surface, PSMA binding radiopharmaceuticals either remain bound to the cell membrane or are internalized within the cell [6]. For very short-ranged particles, the amount of the radiopharmaceutical internalized compared to surface bound may impact the total energy absorbed by the cell nucleus.

There has been an increasing interest in the utilization of simulation-based models of irradiated cells [7] to gain an understanding of what is occurring on the sub-cellular level. These models often assume cancer cells are spherical for computational simplicity, however real prostate cancer cells exhibit irregularities in shape and size which introduce a substantial challenge to model [7]. Several studies have assessed the validity of using spherical cell models for absorbed dose calculations [7–9], but findings remain conflicting and it still needs to be determined if spherical models are appropriate for absorbed dose calculations.

Furthermore, work is being done to determine the impact of radiopharmaceutical internalization [1, 10–12] and many simulation studies separate the radionuclide source locations into internalized versus externally bound configurations. However, most simulation-based studies do not assess the impact of differing internalization ratios [13, 14]. For example, a study may find higher absorbed nucleus doses when the radiopharmaceutical is internalized within the cell, but this omits the impact of if the ratio of membrane bound activity is much higher than the amount internalized. There are many new PSMA binding radiopharmaceuticals under development [15], likely all with different ratios of externally bound to internalized activity.

Beyond the cell morphology, also of importance is the morphology of the tumour itself. Solid tumours are composed of normoxic, hypoxic, and necrotic regions, corresponding to normal, low, and nearly zero oxygen levels respectively [16]. Hypoxic and necrotic regions may increase absorbed dose heterogeneity within tumours due to decreased blood vessel density and vascularization [17], which make it challenging for the radiopharmaceutical to reach these regions; the presence of hypoxia is known to compromise treatment efficacy and encourage cancer progression [16, 18].

These three factors (cell geometry, the ratio of the radiopharmaceutical membrane bound versus internalized, and the tumour morphology) could impact absorbed doses to prostate cancer cells and ultimately treatment efficacy. Because of the different ranges of ^177^Lu and ^225^Ac particulate emissions, ^177^Lu and ^225^Ac labelled pharmaceuticals may behave differently under different conditions (i.e. different tumour morphologies and cell geometries). To these ends, the goal of this work is firstly to determine the impact of cell geometry, source region (cytoplasm vs. membrane), and ratio of internalization on absorbed doses to simulated prostate cancer cell nuclei and within multicellular prostate cancer tumour models. We then assess the impact of hypoxia and necrosis in the tumour models on the absorbed dose distributions. We use Monte Carlo simulations for all tests, and perform all tests with both ^177^Lu and ^225^Ac and compare the results between the two radioisotopes.

## Methods

Using Geant4 Application for Tomographic Emission (GATE), we first simulated three different single cell models with activity in the cytoplasm (to represent internalized activity) or membrane (to represent surface bound activity) of each model to determine the impact of cell geometry and source location on nucleus absorbed doses as an indicator of deoxyribonucleic acid (DNA) damage. We expanded two of the single cell models to a cell layer because of the extra-cellular range of some particulate emissions, and created nucleus absorbed dose kernels that can be combined in different ratios of cytoplasm to membrane uptake to model radiopharmaceuticals with different uptake patterns. The kernels were applied to 2D multicellular prostate cancer tumour maps (with varying amounts of normoxic, hypoxic, and necrotic regions) to obtain absorbed dose rate maps within the tumours. The impact of cell geometry, internalization ratio, and tumour morphology on the absorbed doses and absorbed dose distributions within the tumours was assessed for both ^177^Lu and ^225^Ac.

### Simulation validation

To validate our simulations, we used MIRDcell V3.10, a multicellular dosimetry tool [19]. MIRDcell analytically calculates the absorbed doses from beta and alpha particles using the continuous slowing down approximation in simple spherical cellular geometries. In MIRDcell, we created two spherical cells with 14 µm diameters directly adjacent to each other, each with a 4 µm radius nucleus within a 7 µm radius cytoplasm. Homogeneous activity of ^225^Ac or ^177^Lu was placed in the cytoplasm of one cell and the self-dose per decay to the nucleus and the cross-dose per decay to the neighbor nucleus was calculated. In GATE, two identical water density cells were defined, and the same absorbed dose values were scored in the nuclei to compare against MIRDcell. Details of the GATE simulations are described below.

### Monte Carlo simulations

The Monte Carlo simulation software GATE version 9.0, based on Geant4 version 10.6.2, was used for all simulations. For simulations of ^225^Ac, the Geant4-DNA *emDNAphysics* physics list was used which allows the simulation of physical interactions of charged particles (e.g. beta and alpha particles) down to very low energies (7.4 eV) in liquid water [20]. The daughter emissions of ^225^Ac were included in the simulations. For ^177^Lu, the *emlivermore* physics list was used which allows the simulation of internal conversion electron and beta particle interactions with matter down to about 250 eV. While the *emlivermore* list enabled us to obtain accurate results with ^177^Lu in less time than the *emDNAphysics* list, it was not satisfactory for ^225^Ac due to not simulating alpha particles [21]. For both ^225^Ac and ^177^Lu, the GATE “ion source” was used, which simulates the full photon spectrum and all charged particle emissions of the simulated radionuclide and any daughter radionuclides.

All simulations were split into 100 jobs which were run in semi-parallel. No cuts (cut-off values below which particles are no longer tracked, which are converted to energies by GATE) were used for ^225^Ac as the *emDNAphysics* list already simulates sufficiently low energies, while cuts of 0.1 µm were used for simulations of ^177^Lu. The source and geometry definitions for the cell geometry comparisons and nucleus absorbed dose kernels are found below in the respective sections.

### Cell geometry comparison

To determine the effect of cell geometry on absorbed doses to the nucleus, we modelled three cell geometries: a spherical, ovoid, and cubic cell. All models had a nucleus, nuclear membrane, cytoplasm, and cell membrane which were water density, and the cells were surrounded by water. The total cell and nucleus volumes were approximately identical in all models (1287 *µ*m^3^ for the total cell and 377 *µ*m^3^ for the nucleus) and were based on cross-sectional areas of human lymph node carcinoma of the prostate (LNCaP) cells derived from human prostate cancer cells [22].

The three different geometries were modelled as such:

- The spherical model consisted of concentric spheres with radii of 4.483 µm and 6.74 µm representing the nucleus and cytoplasm respectively.
- The cubic model had a nucleus and nuclear membrane identical to the spherical model while the cytoplasm was a 10.87 µm length cube.
- The ovoid model had an ellipsoidal nucleus with dimensions of 7.7 µm × 5.4 µm × 17.4 µm and a cytoplasm with dimensions of 12.0 µm × 8.0 µm × 25.6 µm.

**Fig. 1:**
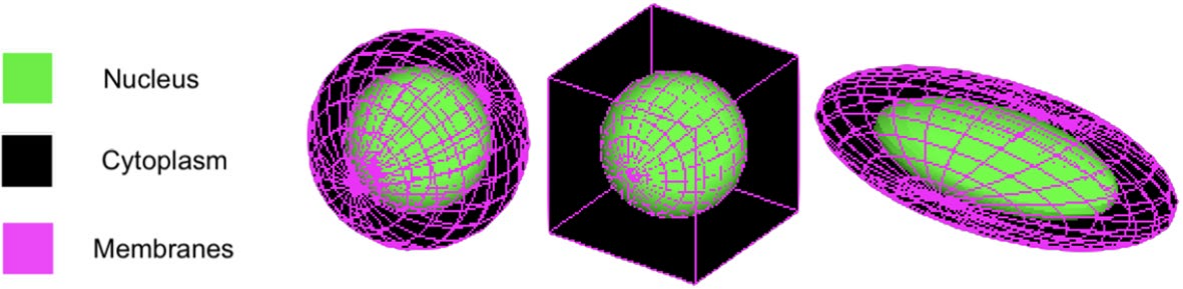
The three cell geometries analyzed in this work, the spherical (left), cubic (middle), and ovoid (right) cell. Images were generated using visualization options in GATE

In all models, the nuclear and cellular membranes were 5 nm thick and enveloped the nucleus or cytoplasm respectively. Figure 1 shows each cell model.

The GATE *DoseActor* tool was attached to each nucleus to score the deposited energy and ultimately calculate the absorbed radiation dose within each voxel. The *DoseActor* outputs 3D images of the deposited energy (“edep”) in units of MeV and the associated uncertainty. The absorbed dose was calculated from the deposited energy outputs as described below in Sect. "S-value calculation". For each single cell geometry, the *DoseActor* had a voxel size of 0.2 µm × 0.2 µm × 0.2 µm. 1 × 10^9^ and 1 × 10^7^ primaries were simulated for ^177^Lu and ^225^Ac respectively. Simulations with the *emDNAphysics* list are more time intensive (taking approximately 6 days to simulate 1 × 10^7^ primaries of ^225^Ac, compared to approximately 30 min to simulate 1 × 10^9^ primaries of ^177^Lu with the *emlivermore* list) and 1 × 10^7^ primaries was the maximum amount that was feasible with ^225^Ac.

### Nucleus absorbed dose kernels

While human tissue consists of layers or clusters of cells, simulating thousands of cells with GATE is computationally intensive and time consuming. To best replicate this biological environment with our simulation constraints, we created a 2D multi-purpose nucleus absorbed dose kernel which describes the absorbed dose deposited in each cell nucleus in a layer of cells when activity is present in the cell at the center of the grid. This kernel can be convolved with simulated or real tissue activity images to move from activity values to absorbed dose rate values, enabling dosimetry and analysis of tissue heterogeneity. We created kernels for both ^177^Lu and ^225^Ac, both source locations, and with both spherical and cubic cell geometries to investigate the effect of cell clustering (i.e. the presence of gaps in between the cells) on kernel values.

**Fig. 2:**
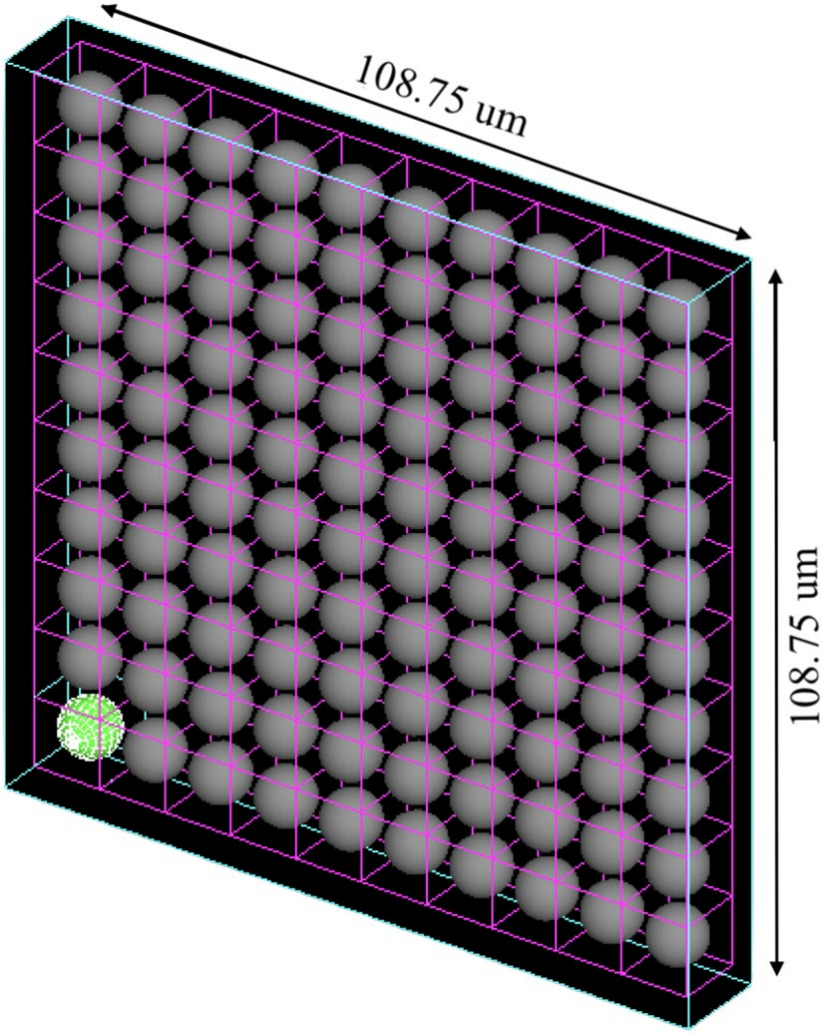
The ^225^Ac cell distribution for the cubic cell kernel modelled in GATE. The cell with the green nucleus is the source cell. The ^177^Lu cubic kernel was identical, however was 56 × 56 cells instead of 10 × 10

To create the kernels, cells were replicated in the positive X and Y directions every cell length (in this case 13.498 µm or 10.875 µm for the spherical and cubic cells respectively) using a Bash script. This created a layer of cells with the cell containing activity placed at the left most bottom quadrant (see Fig. 2). To reduce the simulation memory, each of the replicated cells only contained a nucleus and a cytoplasm; however 10 µm was added to the replicated cytoplasm to account for the missing membranes and maintain consistent cellular spacing. The original source cell had all four cell regions (nucleus, nuclear membrane, cytoplasm, and cellular membrane). The areas in between the cells were water density.

**Fig. 3:**
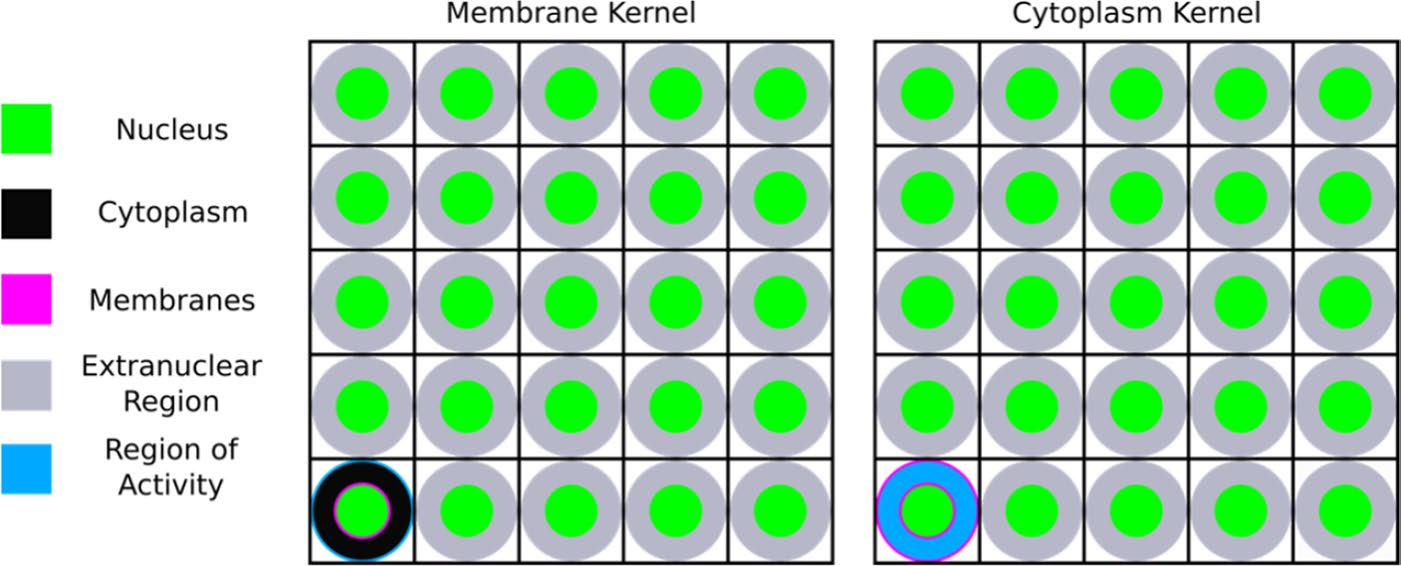
A 2D, 5 cell × 5 cell subset of the input for the simulation of the spherical absorbed dose kernels (not to scale) with one full cell geometry in the bottom left quadrant. All other cells contain only the cell nucleus and extranuclear region. The white gaps are water density and the black lines represent the pixel boundaries

For each radionuclide and geometry (spherical and cubic), two kernels were created (see Fig. 3): i) when activity was placed homogenously in the cytoplasm of the central cell to simulate radiopharmaceutical internalization, and ii) when activity was placed on the cell membrane to simulate a radiopharmaceutical that is cell surface bound. The GATE *DoseActor* was attached to each nucleus in the cellular grid and was 1D, meaning the “edep” output file ascribed a singular value corresponding to the total energy deposited in each cell nucleus (compared to the single cell simulations where the *DoseActor* was 3D, resulting in a 3D matrix as output). The pixel size was one cell size (see Table 1) to enable convolution with cellular activity maps. Despite this, because the *DoseActor* is attached to the nucleus it only describes the energy deposited in the nucleus. 1 × 10^8^ and 1 × 10^6^ primaries were simulated for ^177^Lu and ^225^Ac respectively.

**Table 1:** The final dimensions and lengths of all kernels post-processing

Cell geometry	Pixel size (µm)	^225^Ac		^177^Lu	
		Matrix size	Length (µm)	Matrix size	Length (µm)
Spherical	13.5 × 13.5	15 × 15	202.5 × 202.5	81 × 81	1093.3 × 1093.3
Cubic	10.9 × 10.9	19 × 19	206.6 × 206.6	111 × 111	1207.1 × 1207.1

Using Python, the GATE *DoseActor* “edep” outputs for each cell nucleus were combined to create a 2D one-quarter kernel of the deposited energy with the source activity in the bottom left corner. This quarter kernel was transformed appropriately to create a full kernel, with one of the center rows and columns removed to obtain a kernel describing the deposited energy in each cell nucleus from activity in the source region of the center cell. The kernel was converted to units of S-value (absorbed dose per decay) as described in Sect. "S-value calculation". The final sizes of each kernel are presented in Table 1. While simulating only a one-quarter kernel and extrapolating to a full kernel omits the contribution from scattered radiation off of cells outside the quarter region, we believe this effect would be minimal.

While the maximum range of beta particles from ^177^Lu is around 2.0 mm in tissue, the uncertainty in S-value of the kernel at 2.0 mm from the center cell was very high, given the very small pixel sizes of these kernels. Because the X_90_ (the radius of a sphere in which the beta particles have released 90% of their energy) of ^177^Lu in water is approximately 600 µm [23], we did not encompass the full range of beta particles from ^177^Lu (from the kernel center to the end in each direction) as that was not computationally feasible.

To assess the impact of different ratios of internalized versus extracellular bound activity, we combined the kernels in three different ratios of cytoplasm to membrane activity: 3:1, 1:1, and 1:3. This was done using the following equation:

1 $${K}_{final}=\frac{c \cdot {K}_{cytoplasm}+ m\cdot {K}_{membrane}}{c+m}$$

where *c* and *m* are the ratios of activity in the cytoplasm to membrane respectively, and *K*_*cytoplasm*_ and *K*_*membrane*_ are the cytoplasm and membrane kernels respectively. The ratios of 3:1, 1:1, and 1:3 were chosen to represent the difference between radiopharmaceuticals with cytoplasm dominant uptake, membrane dominant uptake, or equal uptake. However, these kernels can be summed in any ratio to match the internalization pattern of a specific radiopharmaceutical that can be measured with internalization assays.

### S-value calculation

S-values were calculated according to the MIRD schema, which defines the S-value as the mean absorbed dose to a target region per radioactive decay in a source region [24]. The “edep” matrices obtained from the GATE *DoseActor* were used to calculate all S-values. For the single cell simulations, the deposited energy in each voxel of the matrix was summed, converted from MeV to Joules, and divided by the nucleus mass and number of simulated primaries (also known as decays or histories) to obtain the total S-value in the cell nucleus from the source region in units of Gy/Bq/s as shown in Eq. 2. The same calculation was performed for each pixel of the extended nucleus absorbed dose kernels, however no summation was necessary as each pixel already represented the total energy deposited in each nucleus.

2 $${S}_{value}= \frac{\sum_{i=1}^{n}{E}_{i}}{{M}_{nucleus}\cdot {N}_{primaries}}$$

where *E*_*i*_ is the energy deposited in the *i*th voxel, *n* is the total number of voxels in the cell nucleus, *M*_*nucleus*_ is the mass of the nucleus, and *N*_*primaries*_ is the number of primaries simulated with GATE.

### 2D tumour maps

2D multicellular tumour maps containing normal (healthy) cells, blood vessel cells, normoxic cancer cells, hypoxic cancer cells, and necrotic cancer cells were used to study the impact of hypoxia and necrosis on the absorbed dose distributions. These maps were created by Ahn et al., and a description of their creation and validation is described in the cited work [25]. Three distinct tumour morphologies were used in this study, composing mostly normoxic cancer cells, hypoxic cancer cells, or necrotic cancer cells. Two sizes of each morphology were studied, corresponding to approximately 5.5 mm and 10 mm in diameter.

To convert the tumour grids to activity maps, we assumed that the activity uptake within each cell depended on its oxygenation level, as the amount of oxygen was assumed to correlate to blood vessel availability and the ability for the radiopharmaceutical to reach the cell. According to Ahn et al., cells in the tumour model became hypoxic or necrotic when they had oxygen levels of 0.08 to 0.5% and < 0.08% respectively [25] and we assumed a value of 6% O_2_ for normoxic cells based on typical tissue oxygen levels [18, 26]. Based on these numbers, we assumed 91.2%, 7.6%, or 1.2% of the activity of each radionuclide went to each normoxic, hypoxic, and necrotic cell respectively. This was implemented by assigning 91.2 Bq, 7.6 Bq, or 1.2 Bq to each pixel of the tumour map depending on the cancer cell in that pixel, and then normalizing each pixel by the tumour’s total activity (obtained by summing the activity in each pixel) to ensure a total activity of 1 Bq per tumour. This enabled absorbed dose rate maps to be normalized in units of Gy/Bq/s. No activity was assumed to accumulate in the normal cells or blood vessel cells, under the assumption that there would be no uptake in off-target cells.

The summed 3:1, 1:1, and 1:3 cytoplasm to membrane ratio nucleus S-value kernels were convolved with the tumour activity maps to produce a 2D image of the absorbed dose rate per unit activity (DR/A) within the tumours in units of Gy/Bq/s. To perform the convolution, we used the ndimage.convolve function available in the Python SciPy package [27].

### Data analysis

For each tumour morphology and size, the mean DR/A to each cell type (normal cells, blood vessel cells, and the three cancer cell types) was calculated. Dose-volume histograms (DVHs) for each cancer cell type and tumour morphology were created as a measure of absorbed dose heterogeneity within the tumours.

To assess the differences between the three different internalization ratios and the two different cell geometries used in the kernels on the tumour DR/A maps, a statistical analysis of variance (ANOVA) test was done on the mean DR/A to each cell type for each morphology using the scipy.stats function by SciPy in Python [27]. Two sets of ANOVA tests were done: the first compared the kernel geometries (the mean DR/A across all cells after the tumours were convolved with the kernels of different geometries, while the internalization ratio was kept the same) and the second compared the internalization ratios (the mean DR/A across all cells after the tumours were convolved with the kernels of different internalization ratios, while the geometry was kept the same). These tests were evaluated for both ^177^Lu and ^225^Ac.

## Results

### Simulation validation

Table 2 summarizes the results of our simulation validation with MIRDcell. We observed that differences in self-doses for ^177^Lu and ^225^Ac were within 1% and 4%, respectively. Differences in cross-doses were within 5% and 3%, respectively.

**Table 2:** The self-dose and cross-dose S-values from MIRDcell and our GATE simulations in units of mGy/Bq/s

	^177^Lu			^225^Ac		
	Value from MIRDcell	Value from GATE	Percent difference (%)	Value from MIRDcell	Value from GATE	Percent difference (%)
Self S-value	0.307	0.305	− 0.75	40.6	38.98	− 4.14
Cross S-value	0.038	0.0400	− 5.04	6.6	6.44	− 2.56

### Cell geometry comparison

Table 3 shows the S-values from the source region (cytoplasm or cell membrane) to the cell nucleus for all combinations of cell models (spherical, cubic, and ovoid) and radionuclides (^177^Lu and ^225^Ac). We also include the S-value ratio between ^225^Ac and ^177^Lu and the percent difference in the S-value to the same regions between each cell geometry. A difference in cell geometry caused percent differences in S-value ranging from 0.8% to 13.4%. The ovoid cell model had the largest difference in nucleus absorbed doses from the other two cell models, likely because of the variation in nucleus shape (ovoid vs. spherical). Nucleus doses were consistently highest within spherical cells and lowest within ovoid cells, although differences were relatively small.

**Table 3:** The S-values in units of Gy/Bq/s for ^177^Lu and ^225^Ac from the source (column) to the target nucleus (row) for the different cell geometries

Cell Shape	S-Value (Gy/Bq/s)				Ratio ^225^Ac to ^177^Lu	
	Cytoplasm	Cell membrane	Cytoplasm	Cell membrane	Cytoplasm	Cell membrane
Spherical	3.06 × 10^–4^	1.91 × 10^–4^	3.91 × 10^–2^	2.56 × 10^–2^	127.94	134.12
Cubic	2.86 × 10^–4^	1.85 × 10^–4^	3.73 × 10^–2^	2.54 × 10^–2^	127.96	133.34
Ovoid	2.68 × 10^–4^	1.80 × 10^–4^	3.42 × 10^–2^	2.39 × 10^–2^	130.12	137.81
Comparison	Percent Difference (%)					
Spherical vs. Cubic	6.8	3.2	4.7	0.8		
Cubic vs. Ovoid	6.5	2.7	8.7	6.1		
Spherical vs. Ovoid	13.2	6.0	13.4	6.9		

S-values were 39% to 46% higher for ^177^Lu and 35% to 42% higher for ^225^Ac when the radionuclide was internalized in the cytoplasm compared to when bound on the cell membrane. S-values from ^225^Ac were 127–138 times higher than those from ^177^Lu, with the ratio increasing when the radionuclide was membrane bound.

### Nucleus absorbed dose kernels

**Fig. 4:**
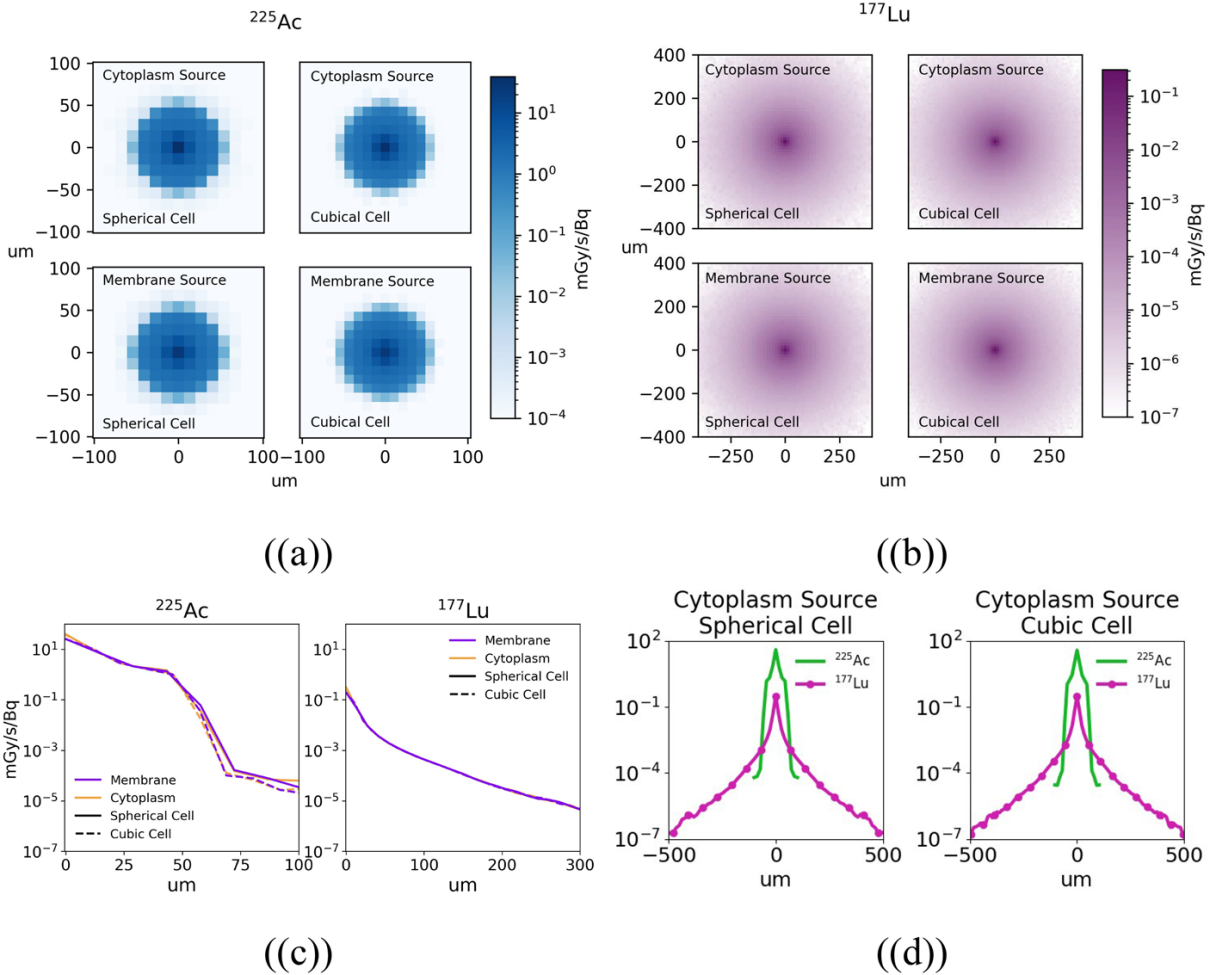
**a** All ^225^Ac kernels. **b** All ^177^Lu kernels. **c** Profile view through the center of the kernels intended to show the differences between the spherical and cubic kernels. One half of the symmetrical kernel is shown. The spherical kernels are solid lines and the cubic kernels are dashed lines. **d** Profile view through the center of the cytoplasm source kernels intended to show the differences between ^177^Lu and ^225^Ac. Each pixel represents one cell

The 2D distributions and profiles of the nucleus absorbed dose kernels are shown in Fig. 4. Visually, there is very little difference between the spherical and cubic cell kernels. The kernels show that ^225^Ac deposits its energy much more rapidly than ^177^Lu, with the deposited energy decreasing by six orders of magnitude after 100 µm compared to four orders of magnitude after 300 µm for ^177^Lu. Plots of the percent uncertainty for each kernel can be found in the supplementary material.

### Tumour images

**Fig. 5:**
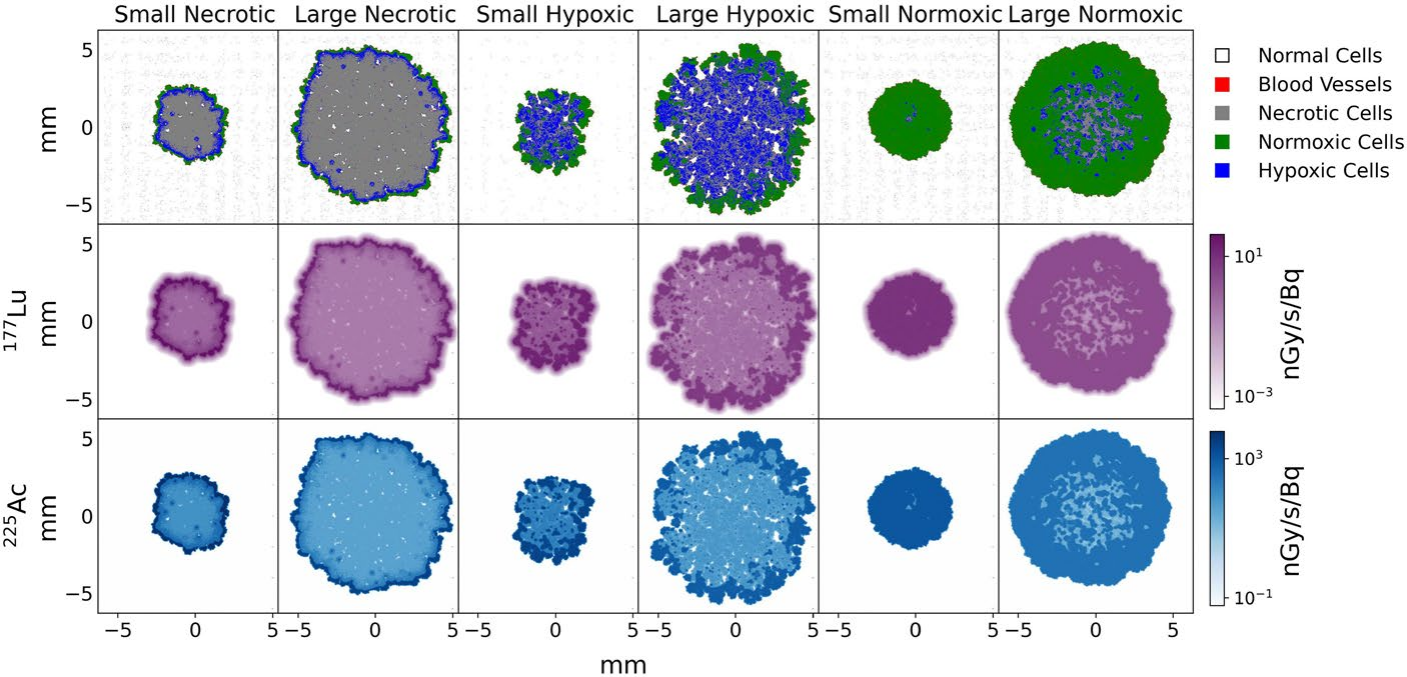
The top row shows the multicellular tumour models, while the subsequent rows show DR/A images in units of nGy/Bq/s after applying the ^177^Lu (middle) and ^225^Ac (bottom) spherical nucleus kernels (with a 3:1 cytoplasm to membrane internalization ratios)

Figure 5 shows the multicellular tumour models and the respective absorbed dose rate per unit activity (DR/A) maps after convolution with the ^177^Lu or ^225^Ac nucleus kernels. Table 4 shows the number of cells of each cell type present in each tumour morphology. For simplicity, all the following images and tables shown are the 3:1 cytoplasm to membrane radiopharmaceutical internalization ratio and created using the spherical cell kernels if not otherwise stated.

**Table 4:** The number of cells of each type in each tumour morphology

Tumour type	Cell type				
	Normal	Blood Vessels	Normoxic	Hypoxic	Necrotic
S. Normoxic	2,000,580	83,602	110,881	787	474
L. Normoxic	1,694,873	74,963	355,821	35,792	34,875
S. Hypoxic	2,069,344	16,893	45,974	45,852	18,261
L. Hypoxic	1,751,534	15,052	127,030	208,589	94,119
S. Necrotic	2,010,349	82,254	17,862	25,647	60,212
L. Necrotic	1,699,304	69,520	38,443	59,505	329,552

**Fig. 6:**
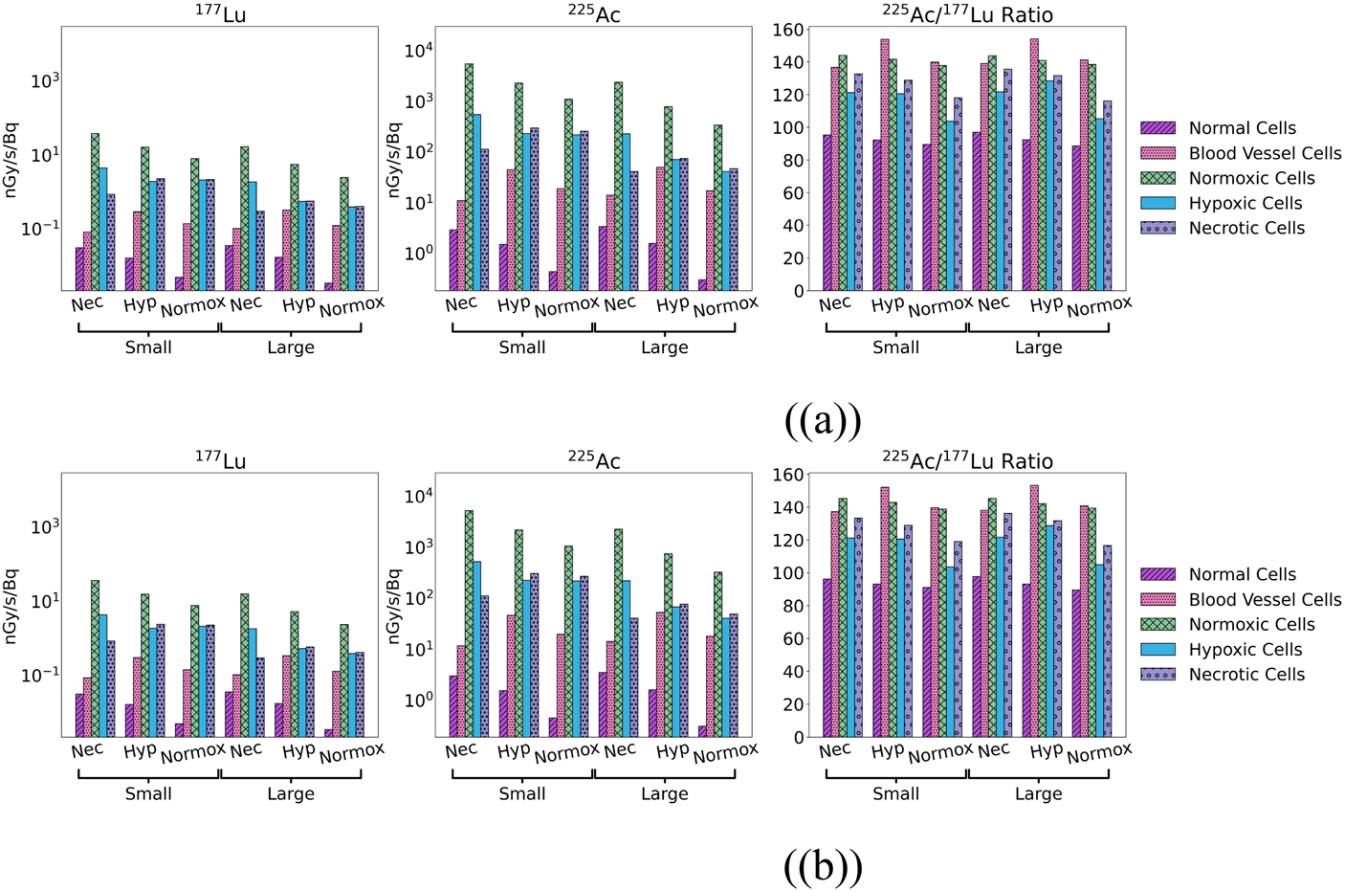
Mean DR/A values (nGy/Bq/s) to all cell types for each tumour type (3:1 cytoplasm to membrane radiopharmaceutical internalization ratio and **a**: spherical and **b**: cubic cell kernel) for ^177^Lu (left column) and ^225^Ac (middle column). The right column shows the ratio of the DR/A from ^225^Ac to ^177^Lu for each cell type and tumour morphology

Figure 6 shows the mean DR/A to each cell type for all tumour morphologies and the ratio of DR/A values between ^225^Ac and ^177^Lu. The mean DR/A to a given cell type varies amongst the different morphologies but stays relatively consistent between the small and large tumours of a given morphology. DR/A values from ^225^Ac are around 100–150 times higher than those from ^177^Lu depending on cell type, with the ratio being on average lowest in normal cells and highest in blood vessel cells.

Table 5 shows the mean DR/A to each cell type averaged after convolution with each nucleus absorbed dose kernel (the spherical and cubic kernels and the 3:1, 1:1, and 1:3 internalization ratio kernels for each geometry) and the associated standard deviation. This is presented for each tumour morphology. The tumour morphology makes a visible difference in the DR/A to each cell type. In contrast, the standard deviation never exceeds 4% of the mean, indicating that the variation in DR/A between each kernel is small; the choice of nucleus absorbed dose kernel (in other words the choice of cell geometry and internalization ratio) makes a small impact on the absorbed dose rates.

**Table 5:** The mean DR/A and associated standard deviation to each cell type and each tumour morphology from all the nucleus absorbed dose kernels (the spherical and cubic kernels and the 3:1, 1:1, and 1:3 internalization ratio kernels for each geometry) for ^177^Lu and ^225^Ac for all tumour morphologies

Mean DR/A (nGy/Bq/s)						
	Cell type					
Tumour type	Normal	Blood Vessels	Normoxic	Hypoxic	Necrotic	Total
^177^Lu						
S. Normoxic	0.005 ± 6 × 10^–5^	0.08 ± 2 × 10^–3^	7.7 ± 0.2	2.1 ± 0.01	2.2 ± 0.04	7.60 ± 0.3
L. Normoxic	0.0034 ± 3 × 10^–5^	0.13 ± 3 × 10^–3^	2.3 ± 0.08	0.4 ± 6 × 10^–3^	0.42 ± 6 × 10^–3^	2.01 ± 0.06
S. Hypoxic	0.026 ± 2 × 10^–4^	0.30 ± 6 × 10^–3^	15.6 ± 0.6	1.9 ± 0.05	2.3 ± 0.04	7.58 ± 0.2
L. Hypoxic	0.017 ± 2 × 10^–4^	0.33 ± 8 × 10^–3^	5.3 ± 0.2	0.5 ± 0.02	0.58 ± 9 × 10^–3^	1.95 ± 0.06
S. Necrotic	0.031 ± 3 × 10^–4^	0.084 ± 2 × 10^–3^	36.0 ± 1.4	4.3 ± 0.1	0.8 ± 0.01	7.78 ± 0.3
L. Necrotic	0.035 ± 4 × 10^–4^	0.10 ± 1 × 10^–3^	15.7 ± 0.6	1.8 ± 0.05	0.3 ± 6 × 10^–3^	1.89 ± 0.06
^225^Ac						
S. Normoxic	0.45 ± 0.01	19.4 ± 0.4	1062.0 ± 28.4	215.9 ± 1.8	263.3 ± 7.0	1052.7 ± 28.0
L. Normoxic	0.31 ± 0.01	17.7 ± 0.4	324.9 ± 8.8	40.4 ± 0.6	48.3 ± 0.9	278.4 ± 7.3
S. Hypoxic	1.5 ± 0.03	45.6 ± 0.7	2193.2 ± 65.6	224.2 ± 5.3	300.0 ± 5.5	1059.4 ± 28.8
L. Hypoxic	1.6 ± 0.02	51.4 ± 1.2	752.2 ± 22.4	68.5 ± 1.8	76.1 ± 1.4	272.3 ± 7.3
S. Necrotic	3.0 ± 0.05	11.4 ± 0.4	5231.0 ± 161.4	521.7 ± 13.6	111.3 ± 1.6	1096.3 ± 30.6
L. Necrotic	3.4 ± 0.05	14.2 ± 0.1	2276.8 ± 66.4	218.9 ± 5.5	40.3 ± 0.7	266.1 ± 7.4

The mean DR/A to all cancer cells for each morphology is also presented in Table 5. Smaller tumours had higher DR/A values. The DR/A from ^225^Ac was 138.6 and 138.4 times higher than ^177^Lu for the small and large normoxic tumours respectively, 139.8 and 139.6 times higher than ^177^Lu for the small and large hypoxic tumours respectively, and 141.3 and 141.1 times higher than ^177^Lu for the small and large necrotic tumours respectively. Despite there being variation in the mean DR/A ratio to each cell type from ^225^Ac and ^177^Lu (see Figs. 6(a) and 6(b)), the ratio of the total tumour DR/A was relatively consistent amongst all tumours.

**Fig. 7:**
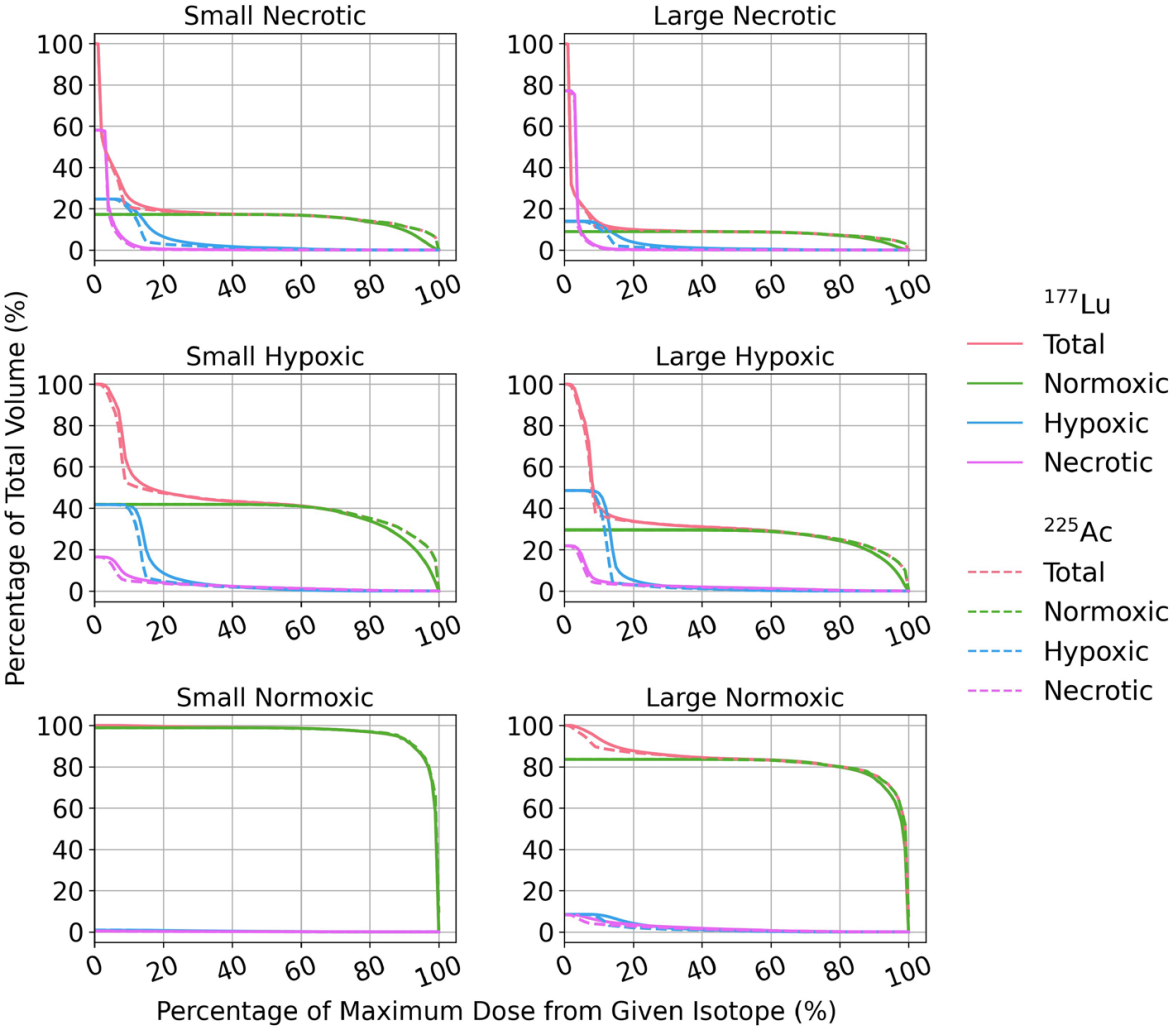
DVHs for each tumour morphology and cancer cell type for the spherical cell geometry and 3:1 membrane to cytoplasm internalization ratio as a percentage of the total absorbed dose

Figure 7 shows DVHs for each tumour morphology, cancer cell type, and radionuclide. The heterogeneity of the absorbed dose distributions was dependent on the tumour morphology and size, with larger tumours exhibiting more heterogeneity. Normoxic tumours had relatively homogeneous dose distributions, but the necrotic and hypoxic tumours exhibited very heterogeneous absorbed dose distributions. Most notably, only 10–20% of the necrotic tumour volume received the maximum absorbed dose. In these tumours, most of the absorbed dose was delivered to regions densely populated with normoxic cells, which were predominantly on the tumour periphery with up to 100 times less dose delivered to the central regions in some cases (see Fig. 5). It should be noted that tumours in a patient may not have the same cell patterns observed here and could have significantly different cell distributions. The cell geometry and internalization ratio made almost no difference on the tumour heterogeneity, in contrast to the tumour morphology. Absorbed dose distributions from ^177^Lu were more homogeneous than ^225^Ac as expected due to the larger range of beta particles, although the difference was relatively small.

### Statistical ANOVA tests

All tests resulted in a failure to reject the null hypothesis, so we cannot say that differences found in the mean DR/A between the different cell geometries or internalization ratios are statistically significant. Tables of the *P*-values and F statistics can be found in the supplementary material. Between both ^177^Lu and ^225^Ac, the minimum *P*-value when comparing the cell geometries was 0.9096 (F statistic 0.0135). When comparing the internalization ratios the minimum *P*-value was 0.991 (F statistic 0.0089). We did observe a downward trend in absorbed dose with decreased cytoplasm to membrane uptake and using the cubic instead of spherical kernels (for example, the mean absorbed doses to cancer cells in the small normoxic tumour after convolution with the 3:1, 1:1, and 1:3 ^225^Ac spherical kernels was 1147, 1114, and 1082 nGy/Bq/s respectively, and these values were 1093, 1077, and 1061 nGy/Bq/s after convolution with the same cubic kernels) but the difference was not high enough to be statistically significant.

## Discussion

We simulated three different prostate cancer cell geometries with activities of ^177^Lu or ^225^Ac internalized within the cytoplasm or bound to the cell membrane and compared the differences in S-values to the cell nuclei from each source region. We then simulated cell layers with activity in the cytoplasm or membrane of the bottom left cell and scored the deposited energy in the nucleus of each cell in the layer, which we used to create 2D absorbed dose kernels representing the S-value to each cell nucleus in the layer from activity in each source location of the center cell. The kernels for each source location can be summed in any cytoplasm to membrane uptake ratio to represent the characteristics of a radiopharmaceutical of choice. We applied these kernels to simulated multicellular tumour models of various morphologies with differing levels of cell hypoxia and necrosis to obtain maps of absorbed dose rate per decay. We evaluated absorbed dose rate heterogeneity by creating DVHs, found mean absorbed dose rates per unit activity to each cell type in the tumour models, and performed a statistical analysis to compare the results of different cell geometries and internalization ratios on our results.

We found that cell geometry made a relatively minimal impact on nucleus S-values (< 14% difference between any geometry). This aligns with previous research on alpha and Auger-Meitner emitters; in particular Bastiaannet et al. [7] obtained similar results (a difference of 16.4% based on cell geometry) when comparing spherical cells with irregularly shaped cells segmented using real breast cancer cells and Auger-Meitner emitting radionuclides [7, 8]. However, as Auger-Meitner electrons have different properties to beta and alpha particles these works cannot be directly compared with ours. Regardless, this result contrasts with another study which found a difference in S-value of up to 40% with different cell geometries using ^177^Lu [9]. Nucleus S-values may change with cell volume or nucleus size [28], which was not explored in the scope of this study. S-values may also be more dependent on the clustering behaviour of the cells compared to the geometry as cells tend to change their shapes when closely packed leaving very little gaps between cells [7]. However, we found no statistically significant difference between the spherical and cubic cell kernels when convolved with the tumour models despite the clustering differences between spheres and cubes (cubic cells leave no gaps between the cells).

We also found that S-values were higher when the radiopharmaceutical was internalized in the cytoplasm compared to bound to the cell membrane which has been verified by others [9, 13]. This would suggest that using radiopharmaceuticals with a larger internalization ratio would result in higher nucleus doses and better cancer cell eradication. This has been a common thought for decades, as it has traditionally been thought that RPT with receptor agonists (which activate receptors) are superior to antagonists (which bind to receptors but do not activate them) as agonists are internalized after receptor binding [11]. However, recent research has shown using antagonists may result in better RPT outcomes despite the reduced internalization, seemingly due to greater cell binding [1, 11, 12], which highlights the importance of the ratio of internalized to extracellular bound activity.

Despite obtaining higher nucleus S-values from internalized activity, we found no statistically significant difference in the mean DR/A to tumour cells based on the internalization ratio. We did notice a downward trend in absorbed doses when the ratio of internalized to extracellular bound activity was lower, but it was not statistically significant. It should be noted that these results assume that the nucleus absorbed dose is the only effect impacting cell survival. Cytoplasm irradiation has also been shown to influence cell survival and DNA synthesis due to both direct and indirect radiation effects (e.g. the bystander effect [29]) although the specific mechanisms behind this are not fully understood. Furthermore, radiopharmaceuticals may have uptake in the cell nucleus or other regions not studied here, which could be especially of impact for radionuclides like Ac that have short-range particle emissions.

A potentially unexpected observation was the fast reduction in the ^177^Lu absorbed dose per decay with distance seen in the nucleus absorbed dose kernels. Typically, ^177^Lu absorbed dose kernels are thought to exhibit a gradual decline in absorbed dose with distance. Most kernels feature considerably larger pixel sizes (ranging from 0.1 mm up to 5 mm) as these values are more relevant for clinical dosimetry [30, 31]. As our pixel sizes are approximately 0.01 mm^2^, we can capture information in the 0–100 µm range that kernels with larger pixel sizes omit. Despite the maximum beta particle range of ^177^Lu being relatively large (2.0 mm), a substantial portion of the absorbed dose may be localized very close to the source which is not considered with large pixel size kernels. This is less surprising considering the combined mean range of internal conversion and beta particles from ^177^Lu is 0.28 mm, considerably smaller than the 2.0 mm maximum [4]. We must note that these kernels do not conform to the standard absorbed dose kernel framework but rather describe the absorbed dose distribution to cell nuclei within a cell layer; however, we believe the distribution is relatively similar to a standard absorbed dose kernel.

Upon applying our kernels to the tumour models, we found heterogeneous absorbed dose distributions across non-normoxic tumour morphologies. This aligns with research finding suboptimal absorbed doses to tissues situated in poorly vascularized regions, such as hypoxic and necrotic tumour microenvironments [32]. Our findings indicate that low oxygen tissues, such as those experiencing hypoxia, may receive suboptimal treatment under the assumption that heterogeneous radiopharmaceutical uptake corresponds to subtumour oxygen levels. However, it must be determined whether lower absorbed doses are seen in these regions due to lower radiopharmaceutical uptake like we assumed here or other factors. The tumour morphology and size had a much larger impact on the DR/A and the absorbed dose heterogeneity than the cell geometry and amount of internalization. There was no statistically significant difference between the tumour cell DR/A values when the spherical versus cubic kernels or the different internalization ratio kernels were applied. This is positive, as future studies at this scale may not be dependent on the cell morphology allowing robust results even if simplifications on the cellular level are made.

^225^Ac has been shown to be more therapeutically effective than ^177^Lu for many reasons, including the higher LET of alpha particles [3, 33]. One group also found a higher uptake of [^225^Ac]Ac-PSMA-617 per unit activity in prostate cancer cells compared to [^177^Lu]LuPSMA-617 [34]. We found mean DR/A values within tumours were 138 to 140 times higher with ^225^Ac compared to ^177^Lu, and dose distributions within tumours from ^225^Ac were more heterogeneous than those from ^177^Lu, although the difference was relatively small (see Fig. 7). This difference would likely increase using the microdosimetry formalism for alpha particles instead of the MIRD formalism like we used here. As low administered activities of ^225^Ac are used in RPT, there are often very few alpha particles traversing each nucleus and some nuclei are not hit at all [35], increasing absorbed dose heterogeneity. In the single cell models, the ^225^Ac to ^177^Lu absorbed dose ratio was higher when the activity was surface bound instead of internalized, likely because the impact of the different particle ranges is more important at a farther distance from the cell nucleus.

Although this work is simulation-based, it opens the door to the generation of absorbed dose rate images using autoradiography of alpha and beta emitters. For example, quantitative alpha or beta particle images of tumours and healthy tissue cross-sections can be obtained using imaging devices such as the ionizing-radiation Quantum Imaging Detector (iQID) [36]. Convolution of our kernels with these autoradiography images could allow the determination of absorbed dose distributions and the study of tissue heterogeneity. This work utilized simulated tumour cross-sections as a substitution for tissue cross-sections, but future studies should move towards autoradiography to obtain more realistic results. To enable and facilitate this research, we will be uploading and sharing our kernels on Zenodo.

We recognize that this study has some limitations. Firstly, due to the very small pixel sizes in our kernels, more simulated decays than are computationally feasible are required to obtain low statistical error throughout the entire kernel. Statistical errors become greater than 10% after approximately 60 µm and 350 µm from the kernel center for ^225^Ac and ^177^Lu respectively. Unfortunately, increasing the number of primaries in the kernels required more time and computational memory than we had access to. As the absorbed dose distribution is arguably more important near the radiation source, we feel this does not impact our results. Secondly, we used the MIRD formalism of absorbed dose, as opposed to microdosimetric techniques, for ^225^Ac calculations. This formalism assumes that many statistically independent radiation events in a single location are required to induce a biological effect [35]. For particles with very high LET such as alpha particles, a single event may be significant enough to render the mean absorbed dose misleading, and therefore this formalism may be less accurate for describing radiation damage at the cellular level [35]. Finally, we assumed that the radiopharmaceutical uptake in each cell was dependent on the oxygenation in that region. While studies have correlated radiopharmaceutical uptake with oxygen concentration [37], this is likely radiopharmaceutical dependent and the exact mechanisms of this are not entirely known at this time.

## Conclusion

We demonstrated that cell geometry has a relatively small impact on absorbed radiation doses to prostate cancer cell nuclei for both ^225^Ac and ^177^Lu, and tumour morphology plays a much more important role in absorbed doses and absorbed dose heterogeneity within tumours than cell geometry and the amount of radiopharmaceutical internalized versus membrane bound. We also generated absorbed dose kernels that enable microdosimetric studies with autoradiography and can be tailored to the internalization ratio of a radiopharmaceutical of interest, which showed that for ^177^Lu a large portion of the absorbed dose may be localized closer to the source than previous kernels have described. Application of our kernels to tumour models of varying morphologies revealed that poorly vascularized tumour regions may receive suboptimal absorbed doses for both ^225^Ac and ^177^Lu.

## Supplementary Information

Additional file 1.

## Data Availability

The datasets generated and/or analysed during the current study are available on Zenodo: https://zenodo.org/records/11359166

## Abbreviations

ANOVA: Statistical analysis of variance
DNA: Deoxyribonucleic acid
DVH: Dose-volume histogram
GATE: Geant4 Application for Tomographic Emission
FDA: Food and Drug Administration
iQID: Ionizing radiation quantum imaging detector
LET: Linear energy transfer
LNCaP: Lymph node carcinoma of the prostate
PSMA: Prostate-specific membrane antigen
RPT: Radiopharmaceutical therapy
SPECT: Single-photon emission computed tomography

## References

[1] Sgouros G, Bodei L, McDevitt MR, Nedrow JR. Radiopharmaceutical therapy in cancer: clinical advances and challenges. Nat Rev Drug Discov. 2020;19(9):589–608. 10.1038/s41573-020-0073-9. 32728208 10.1038/s41573-020-0073-9 PMC7390460

[2] Hennrich U, Eder M. [^177^Lu]Lu-PSMA-617 (PluvictoTM): the first FDA-approved radiotherapeutical for treatment of prostate cancer. Pharmaceuticals. 2022;15(10):1292. 10.3390/ph15101292. 36297404 10.3390/ph15101292 PMC9608311

[3] Ma J, Li L, Liao T, Gong W, Zhang C. Efficacy and safety of ^225^Ac-PSMA-617targeted alpha therapy in metastatic castration-resistant prostate cancer: a systematic review and meta-analysis. Front Oncol. 2022. 10.3389/fonc.2022.796657. 35186737 10.3389/fonc.2022.796657 PMC8852230

[4] Sjogreen Gleisner K, Chouin N, Gabina PM, Cicone F, Gnesin S, Stokke C, Konijnenberg M, Cremonesi M, Verburg FA, Bernhardt P, Eberlein U, Gear J. EANM dosimetry committee recommendations for dosimetry of ^177^Lu-labelled somatostatin-receptor- and PSMA-targeting ligands. Eur J Nucl Med Mol Imaging. 2022;49(6):1778–809. 10.1007/s00259-022-05727-7. 35284969 10.1007/s00259-022-05727-7 PMC9015994

[5] Lee H. Relative efficacy of ^225^Ac-PSMA-617 and ^177^Lu-PSMA-617 in prostate cancer based on subcellular dosimetry. Mol Imaging Radionucl Ther. 2022;31(1):1–6. 10.4274/mirt.galenos.2021.63308. 35114745 10.4274/mirt.galenos.2021.63308 PMC8814544

[6] Sinnes J-P, Bauder-Wust U, Schäfer M, Moon ES, Kopka K, Rösch F. ^68^ga, ^44^sc and ^177^lu-labeled AAZTA5-PSMA-617: synthesis, radiolabeling, stability and cell binding compared to DOTA-PSMA-617 analogues. EJNMMI Radiopharm Chem. 2020. 10.1186/s41181-020-00107-8. 33242189 10.1186/s41181-020-00107-8 PMC7691401

[7] Bastiaannet R, Liatsou I, Hobbs RF, Sgouros G. Large-scale in vitro microdosimetry via live cell microscopy imaging: implications for radiosensitivity and RBE evaluations in alpha-emitter radiopharmaceutical therapy. J Transl Med. 2023. 10.1186/s12967-023-03991-1. 36829143 10.1186/s12967-023-03991-1 PMC9951424

[8] Sefl M, Incerti S, Papamichael G, Emfietzoglou D. Calculation of cellular S-valuesˇ using Geant4-DNA: the effect of cell geometry. Appl Radiat Isot. 2015;104:113–23. 10.1016/j.apradiso.2015.06.027. 26159660 10.1016/j.apradiso.2015.06.027

[9] Tamborino G, De Saint-Hubert M, Struelens L, Seoane DC, Ruigrok EAM, Aerts A, Cappellen WA, Jong M, Konijnenberg MW, Nonnekens J. Cellular dosimetry of [^177^Lu]Lu-DOTA-[Tyr3]octreotate radionuclide therapy: the impact of modeling assumptions on the correlation with in vitro cytotoxicity. EJNMMI Phys. 2020. 10.1186/s40658-020-0276-5. 32040783 10.1186/s40658-020-0276-5 PMC7010903

[10] Borgna F, Haller S, Rodriguez JMM, Ginj M, Grundler PV, Zeevaart JR, Koster U, Schibli R, Meulen NP, Müller C. Combination of terbium-161 with¨ somatostatin receptor antagonists—a potential paradigm shift for the treatment of neuroendocrine neoplasms. Eur J Nucl Med Mol Imaging. 2021;49(4):1113–26. 10.1007/s00259-021-05564-0. 34625828 10.1007/s00259-021-05564-0 PMC8921065

[11] Dalm SU, Nonnekens J, Doeswijk GN, Blois E, Gent DC, Konijnenberg MW, Jong M. Comparison of the therapeutic response to treatment with a ^177^lu-labeled somatostatin receptor agonist and antagonist in preclinical models. J Nucl Med. 2016;57(2):260–5. 10.2967/jnumed.115.167007. 26514177 10.2967/jnumed.115.167007

[12] Fani M, Nicolas GP, Wild D. Somatostatin receptor antagonists for imaging and therapy. J Nucl Med. 2017;58(Supplement 2):61–6. 10.2967/jnumed.116.186783. 10.2967/jnumed.116.186783 28864614

[13] Koniar H, Miller C, Rahmim A, Schaffer P, Uribe C. A GATE simulation study for dosimetry in cancer cell and micrometastasis from the ^225^Ac decay chain. EJNMMI Phys. 2023. 10.1186/s40658-023-00564-5. 37525027 10.1186/s40658-023-00564-5 PMC10390455

[14] Alcocer-Avila ME, Ferreira A, Quinto MA, Morgat C, Hindie E, Champion C. Radiation doses from ^161^Tb and ^177^Lu in single tumour cells and micrometastases. EJNMMI Phys. 2020. 10.1186/s40658-020-00301-2. 32430671 10.1186/s40658-020-00301-2 PMC7237560

[15] Sartor O, Baghian A. Prostate specific membrane antigen binding radiopharmaceuticals: current data and new concepts. Front Med. 2022. 10.3389/fmed.2022.1060922. 10.3389/fmed.2022.1060922 PMC9763319 36561718

[16] Al Tameemi W, Dale TP, Al-Jumaily RMK, Forsyth NR. Hypoxia-modified cancer cell metabolism. Front Cell Dev Biol. 2019. 10.3389/fcell.2019.00004. 30761299 10.3389/fcell.2019.00004 PMC6362613

[17] Horsman MR, Sørensen BS, Busk M, Siemann DW. Therapeutic modification of hypoxia. Clin Oncol. 2021;33(11):492–509. 10.1016/j.clon.2021.08.014. 10.1016/j.clon.2021.08.014 34535359

[18] McKeown SR. Defining normoxia, physoxia and hypoxia in tumours—implications for treatment response. Br J Radiol. 2014;87(1035):20130676. 10.1259/bjr.20130676. 24588669 10.1259/bjr.20130676 PMC4064601

[19] Katugampola S, Wang J, Rosen A, Howell RW. MIRD pamphlet No. 27: MIRDcell V3, a revised software tool for multicellular dosimetry and bioeffect modeling. J Nucl Med. 2022;63(9):1441–9. 10.2967/jnumed.121.263253. 35145016 10.2967/jnumed.121.263253 PMC9454469

[20] Incerti S, Kyriakou I, Bernal MA, Bordage MC, Francis Z, Guatelli S, Ivanchenko V, Karamitros M, Lampe N, Lee SB, Meylan S, Min CH, Shin WG, Nieminen P, Sakata D, Tang N, Villagrasa C, Tran HN, Brown JMC. Geant4-DNA example applications for track structure simulations in liquid water: a report from the Geant4-DNA Project. Med Phys. 2018. 10.1002/mp.13048. 29901835 10.1002/mp.13048

[21] Kyriakou I, Emfietzoglou D, Ivanchenko V, Bordage MC, Guatelli S, Lazarakis P, Tran HN, Incerti S. Microdosimetry of electrons in liquid water using the lowenergy models of Geant4. J Appl Phys. 2017. 10.1063/1.4992076.

[22] Lazar DC, Cho EH, Luttgen MS, Metzner TJ, Uson ML, Torrey M, Gross ME, Kuhn P. Cytometric comparisons between circulating tumor cells from prostate cancer patients and the prostate-tumor-derived LNCaP cell line. Phys Biol. 2012;9(1):016002. 10.1088/1478-3975/9/1/016002. 22306736 10.1088/1478-3975/9/1/016002 PMC3387997

[23] Peer-Firozjaei M, Tajik-Mansoury MA, Geramifar P, Parach AA, Zarifi S. Implementation of dose point kernel (DPK) for dose optimization of ^177^Lu/^90^Y cocktail radionuclides in internal dosimetry. Appl Radiat Isot. 2021;173:109673. 10.1016/j.apradiso.2021.109673. 33812266 10.1016/j.apradiso.2021.109673

[24] Bolch WE, Bouchet LG, Robertson JS, Wessels BW, Siegel JA, Howell RW, et al. MIRD pamphlet no. 17: the dosimetry of nonuniform activity distributions radionuclide S values at the voxel level. J Nucl Med. 1999;40:11–36. 9935083

[25] Ahn HSH, Oloumi Yazdi Y, Wadsworth BJ, Bennewith KL, Rahmim A, Klyuzhin IS. Relating macroscopic PET radiomics features to microscopic tumor phenotypes using a stochastic mathematical model of cellular metabolism and proliferation. Cancers. 2024;16(12):2215. 10.3390/cancers16122215. 38927921 10.3390/cancers16122215 PMC11202285

[26] Osrodek M, Hartman M, Czyz M. Physiologically relevant oxygen concentration (6% O_2_) as an important component of the microenvironment impacting melanoma phenotype and melanoma response to targeted therapeutics in vitro. Int J Mol Sci. 2019;20(17):4203. 10.3390/ijms20174203. 31461993 10.3390/ijms20174203 PMC6747123

[27] Virtanen P, Gommers R, Oliphant TE, Haberland M, Reddy T, Cournapeau D, Burovski E, Peterson P, Weckesser W, Bright J, van der Walt SJ, Brett M, Wilson J, Millman KJ, Mayorov N, Nelson ARJ, Jones E, Kern R, Larson E, Carey CJ, Polat I, Feng Y, Moore EW, Van der Plas J, Laxalde D, Perktold J, Cimrman R, Henriksen I, Quintero EA, Harris CR, Archibald AM, Ribeiro AH, Pedregosa F, van Mulbregt P, SciPy 1.0 Contributors. SciPy 1.0: Fundamental algorithms for scientific computing in Python. Nat Methods. 2020;17:261–72. 10.1038/s41592-019-0686-2. 32015543 10.1038/s41592-019-0686-2 PMC7056644

[28] Kouhkan E, Chegeni N, Hussain A. The effect of nucleus size on the cell dose in targeted radionuclide therapy - a monte carlo study. J Med Signals Sens. 2020;10(2):113–8. 10.4103/jmss.JMSS2119. 32676447 10.4103/jmss.JMSS_21_19 PMC7359958

[29] Zhou H, Hong M, Chai Y, Hei TK. Consequences of cytoplasmic irradiation: Studies from microbeam. J Radiat Res. 2009;50:59–65. 10.1269/jrr.08120s. 10.1269/jrr.08120s PMC3664637 19346686

[30] Graves SA, Flynn RT, Hyer DE. Dose point kernels for 2,174 radionuclides. Med Phys. 2019;46(11):5284–93. 10.1002/mp.13789. 31461537 10.1002/mp.13789 PMC7685392

[31] Kim KM, Lee MS, Suh MS, Selvam HSMS, Tan TH, Cheon GJ, Kang KW, Lee JS. Comparison of voxel s-value methods for personalized voxel-based dosimetry of ^177^Lu-dotatate. Med Phys. 2022;49(3):1888–901. 10.1002/mp.15444. 35014699 10.1002/mp.15444

[32] Birindelli G, Drobnjakovic M, Morath V, Steiger K, D’Alessandria C, Gourni E, Afshar-Oromieh A, Weber W, Rominger A, Eiber M, Shi K. Is hypoxia a factor influencing PSMA-directed radioligand therapy?—an in silico study on the role of chronic hypoxia in prostate cancer. Cancers. 2021;13(14):3429. 10.3390/cancers13143429. 34298642 10.3390/cancers13143429 PMC8307065

[33] Meyer C, Stuparu A, Lueckerath K, Calais J, Czernin J, Slavik R, Dahlbom M. Tandem isotope therapy with ^225^Ac- and ^177^Lu-PSMA-617 in a murine model of prostate cancer. J Nucl Med. 2023;64(11):1772–8. 10.2967/jnumed.123.265433. 37797974 10.2967/jnumed.123.265433 PMC10626377

[34] Current K, Meyer C, Magyar CE, Mona CE, Almajano J, Slavik R, Stuparu AD, Cheng C, Dawson DW, Radu CG, Czernin J, Lueckerath K. Investigating PSMA-targeted radioligand therapy efficacy as a function of cellular PSMA levels and intratumoral PSMA heterogeneity. Clin Cancer Res. 2020;26(12):2946–55. 10.1158/1078-0432.ccr-19-1485. 31932492 10.1158/1078-0432.CCR-19-1485 PMC7299755

[35] Sgouros G, Roeske JC, McDevitt MR, Palm S, Allen BJ, Fisher DR, Brill AB, Song H, Howell RW, Akabani G. MIRD pamphlet No. 22 (abridged): radiobiology and dosimetry of α-particle emitters for targeted radionuclide therapy. J Nucl Med. 2010;51(2):311–28. 10.2967/jnumed.108.058651. 20080889 10.2967/jnumed.108.058651 PMC5680544

[36] Miller BW, Gregory SJ, Fuller ES, Barrett HH, Bradford Barber H, Furenlid LR. The iQID camera: an ionizing-radiation quantum imaging detector. Nucl Instrum Methods Phys Res, Sect A. 2014;767:146–52. 10.1016/j.nima.2014.05.070. 26166921 10.1016/j.nima.2014.05.070 PMC4497505

[37] Mees G, Dierckx R, Vangestel C, Wiele C. Molecular imaging of hypoxia with radiolabelled agents. Eur J Nucl Med Mol Imaging. 2009;36(10):1674–86. 10.1007/s00259-009-1195-9. 19565239 10.1007/s00259-009-1195-9 PMC2758191

