# Supplementary material for: Impact of cell geometry, cellular uptake region, and tumour morphology on 225Ac and 177Lu dose distributions in prostate cancer
Source: EJNMMI Phys. 2024 Nov 21;11:97. doi: 10.1186/s40658-024-00700-9 (PMC11582247; doi:10.1186/s40658-024-00700-9)
Supplement: Supplementary file 1 — Additional file 1. [file 40658_2024_700_MOESM1_ESM.docx]

**Supplementary Material.**


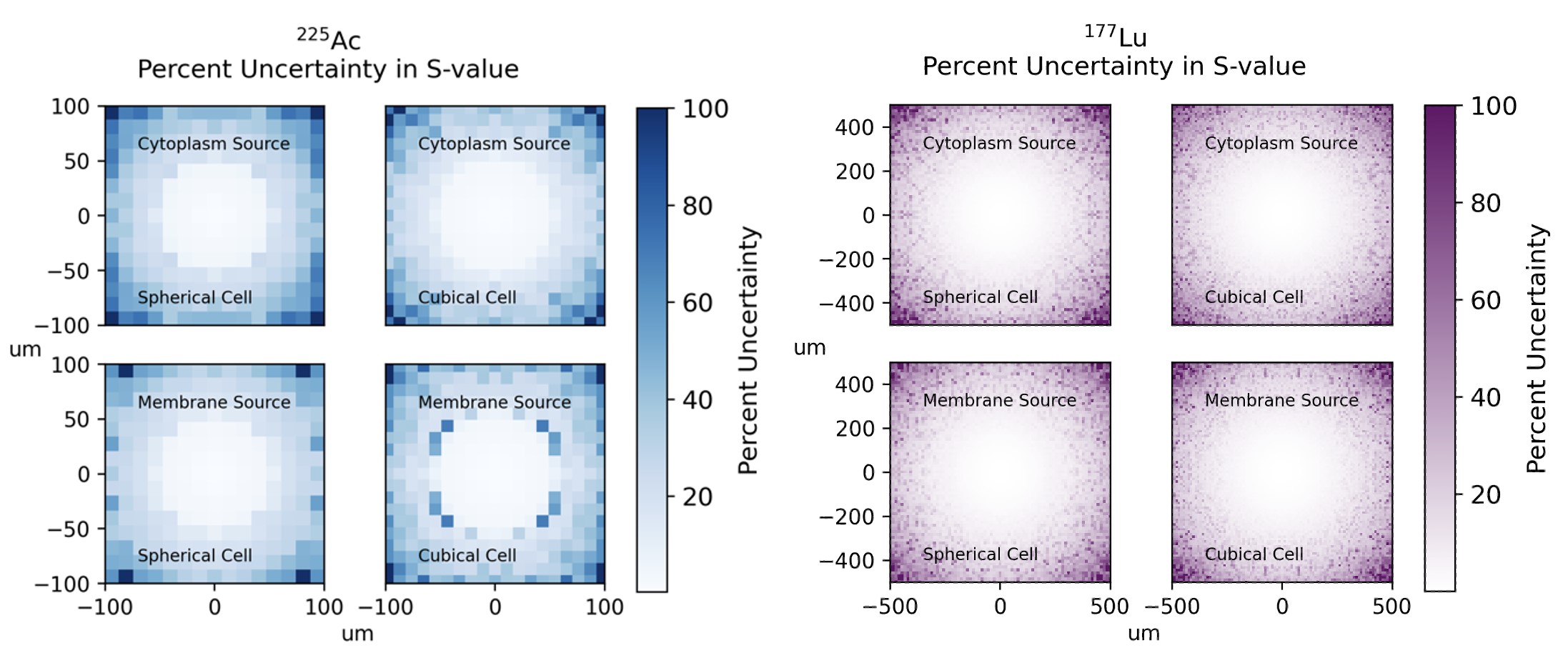


Fig. 1: Percent uncertainty of the ^225^Ac (left) and ^177^Lu (right) kernels.

Table 1: Results of the ANOVA tests comparing the DR/A values after convolution with the different cell geometry kernels. C:M refers to cytoplasm:membrane uptake ratio.

|  |  | ^177^Lu | | | ^225^Ac | |
| --- | --- | --- | --- | --- | --- | --- |
| Cell Type | Internalization  Ratio (C:M) | F Statistic | P-value | F Statistic | | P-value |
| Normal | 3:1  1:1 | 1.9× 10^−3^  1.9× 10^−3^ | 0.97  0.97 | 3.9× 10^−3^  4.9× 10^−3^ | | 0.96  0.95 |
|  | 1:3 | 1.9× 10^−3^ | 0.97 | 6.0× 10^−3^ | | 0.95 |
| Blood Vessel | 3:1  1:1 | 1.3× 10^−1^  1.3× 10^−2^ | 0.91  0.91 | 8.1× 10^−3^  9.5× 10^−3^ | | 0.94  0.93 |
|  | 1:3 | 1.3× 10^−1^ | 0.91 | 1.1× 10^−2^ | | 0.92 |
| Normoxic | 3:1  1:1 | 1.4× 10^−1^  8.1× 10^−3^ | 0.91  0.93 | 1.0× 10^−2^  5.2× 10^−3^ | | 0.92  0.92 |
|  | 1:3 | 3.5× 10^−3^ | 0.95 | 1.9× 10^−3^ | | 0.94 |
| Hypoxic | 3:1  1:1 | 6.8× 10^−3^  3.7× 10^−3^ | 0.94  0.94 | 6.8× 10^−3^  3.2× 10^−3^ | | 0.97  0.94 |
|  | 1:3 | 1.5× 10^−3^ | 0.97 | 8.2× 10^−4^ | | 0.96 |
| Necrotic | 3:1  1:1 | 2.1× 10^−3^  2.6× 10^−2^ | 0.96  0.96 | 2.8× 10^−3^  4.1× 10^−3^ | | 0.98  0.95 |
|  | 1:3 | 3.1× 10^−3^ | 0.96 | 5.7× 10^−3^ | | 0.94 |

Table 2: Results of the ANOVA tests comparing the DR/A values after convolution with the different internalization ratio kernels.

|  |  | ^177^Lu | | | ^225^Ac | |
| --- | --- | --- | --- | --- | --- | --- |
| Cell Type | Shape | F Statistic | P-value | F Statistic | | P-value |
| Normal | spherical  cubic | 3.0× 10^−5^  2.3× 10^−5^ | 1  1 | 2× 10^−6^  9.1× 10^−5^ | | 1 0.99 |
| Blood Vessels | spherical  cubic | 1.7× 10^−4^  1.1× 10^−4^ | 0.99  0.99 | 0  1.2× 10^−4^ | | 1 0.99 |
| Normoxic | spherical  cubic | 9.5× 10^−3^  3.1× 10^−3^ | 0.99  0.99 | 6.7× 10^−3^  1.7× 10^−4^ | | 0.99  0.99 |
| Hypoxic | spherical  cubic | 4.8× 10^−3^  1.5× 10^−3^ | 0.99  0.99 | 5.1× 10^−3^  1.1× 10^−3^ | | 0.99  0.99 |
| Necrotic | spherical  cubic | 1.4× 10^−4^  1.8× 10^−5^ | 0.99  1 | 3.2× 10^−4^  1.0× 10^−6^ | | 0.99  0.1 |
